# Influence Factors and Life Satisfaction of Types of Older Adults’ Daily Time Use Using Latent Profile Analysis

**DOI:** 10.1093/geroni/igaa057.1607

**Published:** 2020-12-16

**Authors:** Joosuk Chae, Seok In Nam, Haesol Won, Juyoung Lee

**Affiliations:** Yonsei University, Seoul, Republic of Korea

## Abstract

The purpose of this study is to divide the daily time use of older adults into types, examine the influence factors of each type, and verify the difference in life satisfaction between types. We used a total of six variables, three variables (essential, compulsory, and leisure time use) for weekdays and the same three variables for weekends. We used data from the sixth wave of the Korea Retirement and Income Study (n=3,993). Latent Profile Analysis was used to classify the older adults’ (aged 65 and over) types of daily time use, resulting in a division into three types: essential activity-centered (16%), leisure-centered (26%), and balanced (58%). The weekend and weekday activities of each group did not differ. Compared with the balanced type, the significant factors of the essential activity-centered type were age (p<.01), work status (p<.001), and chronic disease (p<.001). In addition, the significant factors of the leisure-centered type were age (p<.05), gender (p<.05), subjective health (p<.001), work status (p<.001), and chronic disease (p<.001). Life satisfaction was lowest in the essential activity-centered type and highest in the balanced type; differences between the groups were identified (p<.000). Based on the results, we discuss practical interventions and the development of psychosocial programs for older adults. For the essential activity-centered type, living support is required because they spend more time sleeping and eating. In addition, we proposed the preparation of leisure programs applicable to the needs of older adults’ of the leisure-centered type, since their life satisfaction was lower than the balanced type.

